# Differential expression analysis of human endogenous retroviruses based on ENCODE RNA-seq data

**DOI:** 10.1186/s12920-015-0146-5

**Published:** 2015-11-03

**Authors:** Kerstin Haase, Anja Mösch, Dmitrij Frishman

**Affiliations:** Department of Genome Oriented Bioinformatics, Wissenschaftszentrum Weihenstephan, TU München, Maximus-von-Imhof-Forum 3, Freising, 85354 Germany; Helmholtz Center Munich - German Research Center for Environmental Health (GmbH), Institute of Bioinformatics and Systems Biology, Ingolstädter Landstraße 1, Neuherberg, 85764 Germany; St Petersburg State Polytechnical University, St. Petersburg, 195251 Russia

**Keywords:** Gene expression, Cancer, Next-generation sequencing

## Abstract

**Background:**

Human endogenous retroviruses (HERVs) are flanked by long terminal repeats (LTRs), which possess promoter activity and can therefore influence the expression of neighboring genes. HERV involvement in different types of cancer has already been thoroughly documented. However, so far there has been no systematic study of HERV expression patterns in a multitude of cell types in health and disease. In particular, the publication of the comprehensive ENCODE dataset has already facilitated many gene expression studies, but none so far focusing exclusively on HERVs.

**Results:**

We present a comprehensive differential analysis of HERV expression based on ENCODE Tier 1 and Tier 2 RNA-seq data produced by Cold Spring Harbor Laboratories and the California Institute of Technology. This analysis was conducted for individual HERV loci and for entire HERV families in twelve different cell lines, of which six correspond to the normal condition and the other six represent cancer cell types. Although the principal component analysis revealed that the two groups of cells show distinguishable expression patterns, we were not able to link these differences to one or multiple particular HERV families. Two samples exhibit expression patterns, which are not similar to the corresponding cell lines of the other producing lab. Instead they show signs of cancer formation and expression of the pluripotency marker HERVH, despite being classified as a normal cell line and a differentiated cell, respectively.

**Conclusions:**

Our study demonstrates that ENCODE data are generally comparable between the different contributing labs and that the analysis of HERV elements can provide novel insights into differentiation and disease state of a cell that are easily overlooked when focusing on protein-coding genes. Our findings hint at a change in HERV expression during cancerogenesis.

**Electronic supplementary material:**

The online version of this article (doi:10.1186/s12920-015-0146-5) contains supplementary material, which is available to authorized users.

## Background

Human endogenous retroviruses (HERVs) are remnants of germline infections by exogenous retroviruses that were integrated into the host genome and passed on to the offspring. Estimates place the amount of human DNA that has a retroviral origin at 8 % [1]. Due to the presence of the proviral *pol* gene, which encodes the reverse transcriptase, HERVs can reintegrate copies of themselves in other genomic locations and hence belong to the group of transposable elements. In addition to the *gag*, *pol* and *env* genes, which are counterparts of the original functional virus genes, HERVs also contain long terminal repeat sequences (LTRs) at the 5’ and 3’ end. These LTRs have a strong promoter function, which can increase the transcription level of neighboring genes [2]. As internal viral genes tend to degrade over time due to the absence of evolutionary pressure, many HERV sequences are lacking some, or even all of their ORFs, or contain their fragments. In particular, many solitary LTR sequences can be found in the human genome [3].

Some of the HERV loci in the human genome have been identified as being beneficial to the host. For example, syncytin, encoded by the *env* gene of the HERV-W family is linked to differentiation and morphogenesis of the placental tissue and hypothesized to have immunosuppressive function that supports the maternofetal tolerance [4, 5]. There are also multiple studies linking HERVs to diseases such as multiple sclerosis and schizophrenia [6, 7], although they are mainly based on detecting elevated expression levels of certain HERV families in affected individuals and do not necessarily shed light on causal relationships. HERVs have also been implicated in breast cancer and melanoma [8, 9] where samples of patients contained expressed HERV genes, viral proteins and antibodies for HERV peptides. However, in order to understand the causative mechanisms of HERV involvement in disease, a rigorous tissue specific differential expression analysis of HERV families between disease and healthy states is critically required. Such differential analyses are complicated by the fact that HERVs are repetitive elements spread over the entire genome, which makes mapping of their transcripts to genomic loci particularly challenging. Previous attempts to create an overview of HERV expression patterns in different tissues relied on a specifically designed chip with various captured retroviral pol sequences [10, 11] and were thus limited to the subset of HERV family members that still contain intact pol sequences. In order to increase the number of covered retroviral elements, a more comprehensive approach, capable of identifying the full-length sequences would be required. RNA-Seq has become a method of choice for addressing such problems [12] as it provides precise measurements of transcript levels in the cell and thus makes it possible to map all retroviral elements, both structurally intact and partial, back to their genomic loci.

Over the past ten years the Encyclopedia Of DNA Elements project (ENCODE) [13] has been working on systematic identification of all functional elements in the human genome. With the most recent data release, made available in September 2014, ENCODE incorporates 27 different kinds of experiments, such as Exon Arrays, ChiP-Seq, and RNA-Seq analyses, conducted by seven research labs in order to gather as much information as possible on a standardized group of cell lines. These cell types are subdivided into three tiers depending on their assigned priority. Tier 1, which only contains three different cell types, constitutes the highest priority and has thus the largest number of conducted studies associated with it. All tiers include healthy cell lines as well as cancerous ones, and all data submissions are also subdivided based on the cellular compartments in which measurements were performed. The ENCODE guidelines force submitters to provide at least two biological replicates per experiment for more robust statistical analyses. There are a total of 151 RNA-Seq experiments in the ENCODE summary of the first stage (2007–2012), with 87 of them using small RNA-Seq (as of September 2014).

Since this valuable resource of highly standardized reference data became available, many research groups published studies integrating or comparing the ENCODE data to their own samples or conducting meta-analyses across ENCODE cell lines [14–16]. ENCODE data has also been used in computational studies on gene expression, but as of now transcriptome analyses spanning multiple cell types aim at protein-coding genes or functional regulatory RNAs [17, 18]. Examination of ENCODE RNA-Seq data with regard to HERV expression has either been limited to single cell types or covers HERVs only as a very small subset of the overall analysis [19, 20].

In this work we have comprehensively analyzed ENCODE RNA-Seq data covering all annotated HERV loci in a broad variety of cell lines, disease and developmental stages. We sought to gain an insight into the overall expression patterns of HERV elements and to examine on a large scale if there are measurable differences in HERV activity between cancer and normal cells, as already reported for individual tumor types. Furthermore, a major goal of our study was to assess the consistency of different ENCODE-contributing laboratories with regard to expression values from the same cell lines.

We present the analysis of 25 RNA-Seq samples from ENCODE’s top two priority tiers with regard to the expression of all annotated HERV loci obtained from the HERVd database [21, 22]. We found that there are considerable differences between the expression profiles obtained by single- vs paired-end sequencing, with the former showing lower overall expression. This finding holds true both for HERVs and for housekeeping genes. Apart from the discrepancies stemming from different library designs and two cases probably caused by transformation of the underlying cell lines, we did not observe any striking distinctions between the data provided by the two contributing laboratories. This suggests that ENCODEs quality standards are sufficient to provide a robust basis for comparative analysis.

Upon removal of systematic errors resulting from different sequencing strategies, we were able to identify unusual patterns in HERV expression in two of the analyzed cell lines. Members of the HERV-H family, considered to be a marker for pluripotency and normally seen in embryonic stem cells (ESCs), are strongly overexpressed in the HeLa-S3 cells. Furthermore, one blood cell line, GM12878, shows a HERV expression profile, which is more similar to cancerous blood cells than to another healthy sample, potentially pointing to a cancerous transformation of this cell line. These findings imply that patterns of HERV expression could serve as useful markers in early cancer diagnostics.

## Results

### Differential expression analysis shows strong differences between sequencing technologies

We detected HERV expression in all analyzed samples. The CSHL dataset has on average 120,411,532 mapped fragments, of which 0.73 % are assigned to HERV loci. In Caltech’s paired-end data set, featureCount works with 67,730,782 fragments on average, of which approximately 1.3 % have been mapped to HERVd annotations. The single-end data sets include on average 20,640,141 reads, with 0.42 % of them assigned to HERV loci. A list showing all numbers of mapped fragments per sample as well as the fraction being assigned to HERV loci can be found in Additional file 1.

**Fig. 1:**
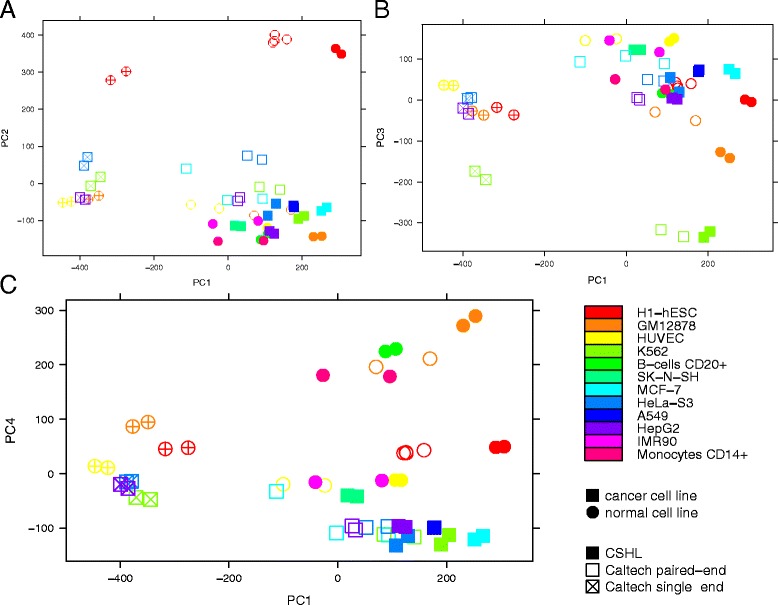
Principal component analysis of normalized HERV expression values in 25 ENCODE RNA-Seq data sets. The first, second, third, and fourth principal components account for 19.06 %, 10.30 %; 5.70 %, and 5.01 % of the variance, respectively. Each data point represents one replicate. Circles: normal cell lines; squares: cancer cells; filled symbols: CSHL samples; empty symbols: Caltech; crosses in the symbols: single-end sequencing. **a** The first component separates single- and paired-end libraries, while the second separates ESC samples from all the others. **b** The third component separates K562 from the other cell types. **c** The fourth component divides samples into normal and cancerous cell types

The principal component analysis of the transformed expression values of all 25 samples (Fig. 1) shows that the strongest differences in HERV expression result from variations of the sequencing technology used. The first principal component (which captures 19.06 % of the total variance) clearly subdivides the Caltech samples into those analyzed by single- and paired-end sequencing. The second and third principal components, accounting for 10.30 % and 5.70 % of the variance, respectively, separate certain cell types from the rest of the datasets. While the second component clusters together all eight embryonic stem cell samples, the third component lets the six K562 samples stand out. Finally, the fourth component (5.01 % of the variance) mostly reflects the differences between normal and cancer cell types, with the exception of the MCF-7 sample from Caltech where one replicate is clearly separated from the other two and overlaps with a normal tissue (HUVEC).

Given that single-end datasets lead to very different results compared to their paired-end counterparts, we decided to exclude them from the further analyses to prevent them from introducing a bias into the expression data. The paired-end data seems to give a better overview of the expression rates. Note that in the GEO summary of the ENCODE Caltech RNA-Seq data, the single-end protocol which is also strand-specific, is described as less reliable for quantification [http://www.ncbi.nlm.nih.gov/geo/query/acc.cgi?acc=GSE33480].

### Housekeeping genes confirm strong differences in ENCODE expression data, especially between different sequencing protocols

**Fig. 2:**
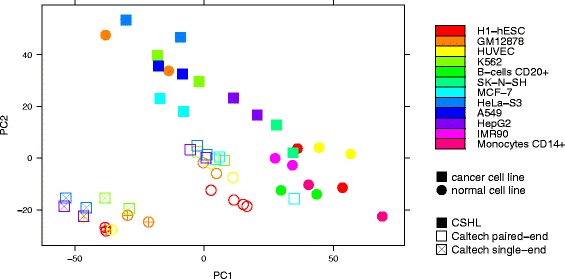
Principal component analysis of normalized expression values of housekeeping genes in 25 ENCODE RNA-Seq data sets. The first and the second principal components account for 30.67 % and 21.33 % of variance, respectively. Circles: normal cell lines; Squares; cancer data sets; Filled symbols: CSHL; Empty symbols: Caltech; crosses in the symbols: single-end sequencing

**Fig. 3:**
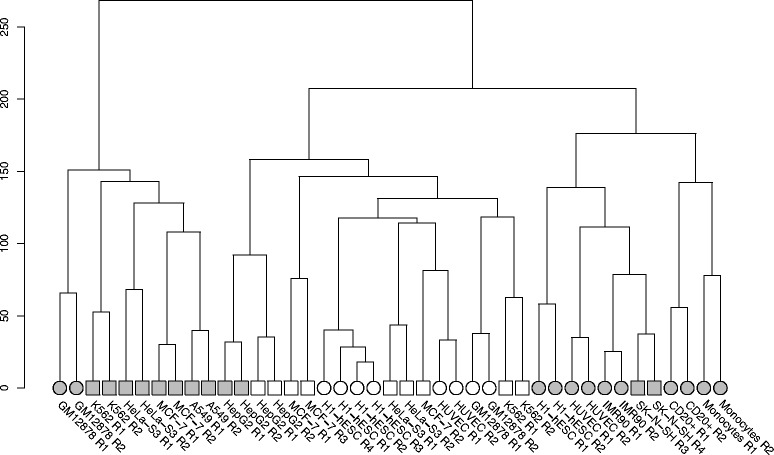
Hierarchical clustering of Euclidean distances between the transformed expression values of housekeeping genes. “Rep*N*” in the dataset identifier represents the replicate number *n*. Normal and cancer cell lines are shown by circles and squares, respectively. Samples analyzed by CSHL are drawn with filled symbols, those from Caltech are drawn with blank symbols

Differences in expression of housekeeping genes were also mostly due to the used library protocols. While the first principal component (accounting for 30.67 % of the variance) primarily divides the CSHL cell lines into cancerous and normal ones (with the exception of CSHL’s GM12878 and a small overlap involving SK-N-SH), the samples provided by Caltech are neatly separated into paired- and single-end protocols (Fig. 2). A hierarchical clustering of housekeeping genes based on the Euclidean distances between their expression vectors yields three subtrees. One of them contains exclusively Caltech and the other two contain only CSHL samples. The two CSHL subtrees separate nearly perfectly cancerous from normal call lines, with the exception of GM12878 and SK-N-SH (Fig. 3).

The observed differences thus do not depend on the chosen transcript family, but are rather indeed an inherent pattern in the ENCODE datasets.

### Two cell lines show behavior atypical for their disease and developmental stage

**Fig. 4:**
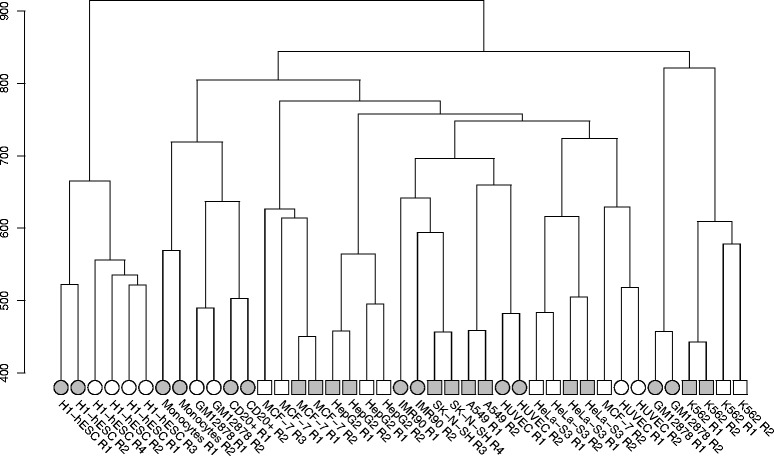
Hierarchical clustering of Euclidean distances between the transformed expression values in paired-end ENCODE data sets. “Rn” in the dataset identifier represents the replicate number *n*. Circles show normal, squares cancer cell lines. Samples analyzed by CSHL are drawn with filled symbols, those from Caltech are drawn with blank symbols

After excluding the single-end Caltech samples, we calculated the Euclidean distances between the transformed expression vectors for all remaining paired-end datasets and performed a hierarchical clustering. As seen in Fig. 4, most replicates are highly similar to each other with regard to HERV expression, with the exception of IMR90 and the second replicate of Caltech’s MCF7 sequencing. ESCs are clearly the most diverse among the differentiated cell types, serving as an outgroup. Particularly striking is the clustering of CSHL’s GM12878 replicates. While the same cell line, analyzed by Caltech, is branched together with two other healthy blood cell types, GM12878’s expression vectors determined at CSHL cluster with all four examined blood cancer samples (K562).

**Fig. 5:**
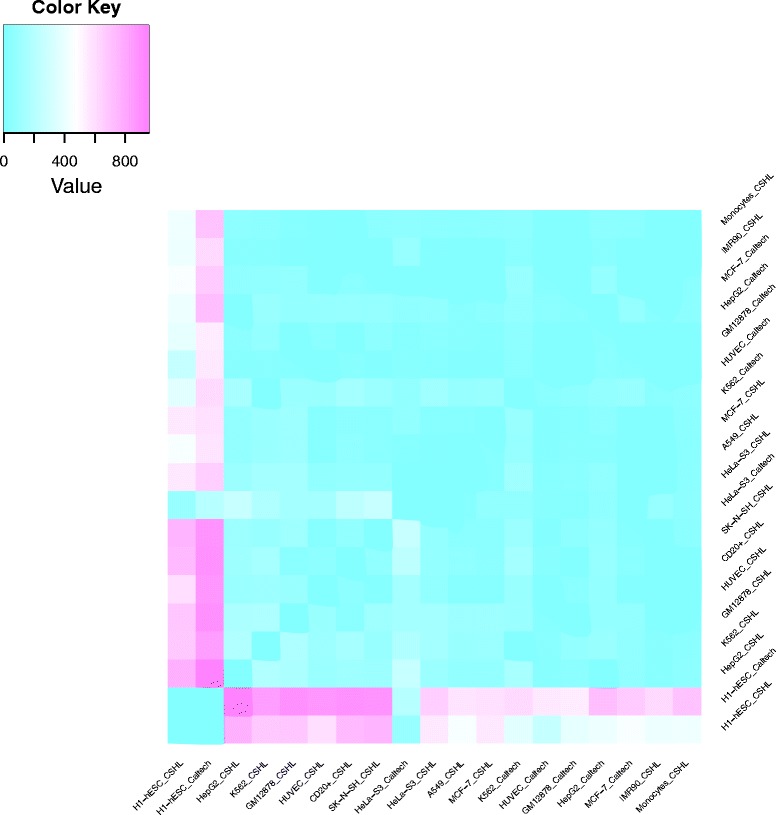
Heatmap of significantly differentially expressed HERV loci. Number of significantly (|log_2_ fold change| at least one, adjusted p-value < 0.001) differentially expressed HERV loci in pairwise comparisons. Ice blue fields show comparisons yielding none or very few differentially expressed loci, hot pink represents large numbers (up to 956)

In addition to the global clustering presented above we also compared every condition against every other condition in a pairwise fashion, leading to 171 differential expression analyses. Following the common practice [23, 24] we defined significantly differentially expressed HERV loci as those with an absolute value of the logarithmic fold change of at least one and the adjusted p-value smaller than 0.001 (FDR of 0.1 %). The number of identified loci varied depending on the compared samples (Fig. 5). Pairwise analyses involving embryonic stem cells led to the largest number of differences with up to 956 significant loci (Caltech H1-hESC vs. CSHL HepG2). The smallest number of significantly differentially expressed loci between two different cell types is three and occurs three times (Caltech and CSHL HUVEC vs. IMR90 and Caltech MCF-7 vs. CSHL HeLa-S3). While the largest numbers of differentially expressed HERVs are seen when ESCs are compared to the other cell types, it is remarkable that Caltech’s HeLa-S3 line shows very few loci, which are significant in comparison with both H1-hESC samples (see Fig. 5).

### Caltech’s HeLa-S3 cell line shows a strong up-regulation of HERVH, typical for embryonic stem cells

**Fig. 6:**
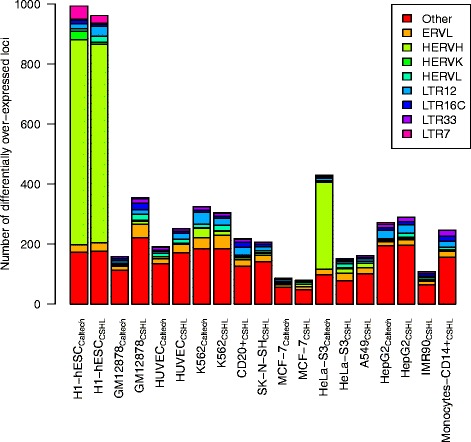
Up-regulated HERV families in different cell types. The plot shows HERV families that are significantly stronger expressed when comparing the indicated cell type against all others. All families that have fewer than 20 members significantly overexpressed in all samples are grouped together in the “other” class

Upon extracting all significantly over-expressed loci per cell type from the pairwise comparisons and grouping them by their family affiliation, we observed characteristic patterns for different samples. The eight most often up-regulated HERV families are four internal and four LTR sequences (Fig. 6). Internal regions that are most overexpressed in the pairwise comparisons are ERVL, a very old endogenous element that is found even outside of primates and shows high similarity to foamy retroviruses [25] and its younger relative HERVL. Additionally, two LTR sequences, LTR16C and LTR33, are also among the eight most often overexpressed families, which belong to the ERVL superfamily. The other two internal families are the pluripotency marker HERVH and the young human endogenous viral element, which is also the most active in terms of expression and transposition, HERVK [26]. The two additional often up-regulated LTR sequences are LTR7, which is a long terminal repeat sequence of HERVH, and LTR12, belonging to the HERV superfamily ERV1.

Overall, the same cell types analyzed by any two laboratories show a similar composition of HERV families, with the exception of GM12878 from CSHL, which exhibits a nearly double amount of significant HERVs compared to its Caltech counterpart. Especially the large number of ERVL members in the CSHL sample is unmatched in the corresponding Caltech cell line. The only other cell types with a similar large number of active HERVL loci is CSHL’s K562 sample, which is also a blood cell type, but contrary to GM12878 cancerous.

Another cell line that exhibits an extremely deviant behavior in the lab comparison is HeLa-S3 from Caltech. It appears to over-express an immense amount of HERVH family members (290 loci), which are only found in low numbers in all other specialized cells. The over-expression is not as strong as in the embryonic stem cells, but is higher than any other number of a single HERV family in all other cell lines (Fig. 6). The difference between cancerous and normal cell lines revealed by the principal component analysis could not be linked to a particular overexpressed HERV family. We were not able to identify any expression patterns separating the six normal from the six cancer cell lines on the basis of individual HERV families.

## Discussion

Based on the comprehensive analysis of 25 RNA-Seq samples from the ENCODE project with regard to their HERV expression we find that datasets created with different sequencing library methods (paired- vs single-end) are not very easily comparable, because single-end samples achieve less coverage. This is expected, as the sequencing technique used by Caltech is strand specific and as such trades quantification against a qualitative analysis.

Although a principal component analysis of the HERV expression patterns in different cell types revealed the possibility to distinguish cancerous from healthy samples based on HERV activity, we could not link this difference to a specific HERV family. However, our study revealed unexpected results regarding GM12878 from CSHL, which showed hints of being a tumorous cell line in two different analyses. First, a hierarchical clustering of all HERV loci expression grouped this cell type with all four K562 (blood cancer) replicates instead of the GM12878 samples from CSHL. Second, the composition of up-regulated HERV families in this sample, when compared to all others, is much more similar to that of K562, especially regarding the strong activity of ERVL. A possible reason for this behavior could be the transformation of an initially normal cell line to a tumorous one prior to experimental measurements. However, this explanation does not seem to be particularly plausible given that ENCODE imposes strict data quality requirements, especially with regard to tier 1 cell lines to which GM12878 belongs. The respective Caltech GM12878 RNA-Seq track has been accessible through the UCSC genome browser [27, 28] since August 2012 and so far no unusual features of this dataset, including a possible progression towards a tumor line, have been reported. It is conceivable that the change in HERV expression detected in our study, which is the first comprehensive investigation of HERV expression in ENCODE samples, occurs very early in the transition from a normal to a cancer cell type and hence remained undetected in studies focusing on protein-coding gene expression, although we were able to detect aberrant behavior hinting at this change when performing PCA on housekeeping genes. Further research is needed to verify this hypothesis, as it implies that unusual HERV expression could serve as an early indication of carcinogenic transformation and thus represent a valuable diagnostic lead.

Another striking finding is the low amount of differentially expressed HERVs when comparing Caltech’s HeLa-S3 sample to the ESCs. The strongest difference between HERV expression in ESCs compared against specialized cell types is the very strong up-regulation of HERVH family members. Because HERVH activity is also high in Caltech’s HeLa cells, unmatched in any of the other differentiated cell types, the difference in expression pattern to ESCs is understandably small. The HERVH family is known to play a vital role in embryonic stem cells. In particular, since they can serve as a marker for pluripotency due to their strong association with binding sites for the pluripotency transcription factors NANOG, OCT4 and SOX2 [29]. Furthermore, it has been suggested that HERVH and its LTR7 can recruit the transcription factors p300 and OCT4 to regulate the transcription of pluripotency-associated transcripts [20]. Intriguingly, Santoni et al. also used ENCODE RNA-Seq data from Caltech to analyze HERVs in hESCs, although they relied on the 2010 data release whereas in this study we utilized the most recent data published in 2012. For comparison with differentiated cells, Santoni et al. also obtained the 2010 data on corresponding HeLa-S3 cells and found that “HERV-H expression is barely detectable in HeLa”, although it was identified when using transient-transfection assays [30]. It is thus apparent that there has been a significant change between the 2010 and 2012 HERV expression data submitted to the ENCODE project by Caltech.

Santoni et al. observed that the HERV expression strength in ESCs diminishes during differentiation. Expression is highest at the undifferentiated N0 stage, still observable during N1 (early initiation), and only barely measurable during N2 (neural progenitor). Thus, a conceivable explanation for the behavior of Caltech’s HeLa-S3 cells would be reprogramming towards pluripotency, although an underlying mechanism for this process remains enigmatic.

## Conclusions

In this study we analyzed the expression of known human endogenous retroviral elements in RNA-Seq samples from the ENCODE project. It is the first examination of sequencing data from a variety of cell types and labs with regard to HERV expression patterns.

We found that all analyzed cell lines have active HERV loci and, by performing differential expression analysis, we identified cell type specific expression patterns. Our analysis also revealed discrepancies between different RNA-Seq datasets: single cell lines showed significantly different expression profiles depending on the laboratory where measurements were conducted. We verified that this finding was not due to the particular kind of transcripts considered, namely HERVs, by repeating the same analysis with housekeeping genes.

Furthermore, in two cases deviant expression patterns were closer to those of a completely different cell type than the same line from a different research institution.

Thus, we believe it is of particular importance to monitor cultured cell lines very closely as small changes to a cell type can lead to major alterations of expression patterns for some non-coding RNAs. While conducting differential expression analysis it might not be sufficient to regard even identical cell lines as comparable. Our analysis of cancer-specific HERV families is further complicated by consideration of distinctly different cancer cell lines. However, PCA shows that there is an informative signal in the expression data differentiating cancer from normal cell lines.

If further investigations confirm that a change in HERV expression patterns is an early sign of cell transformation, it can be utilized as a diagnostic tool to help recognize tumor formation.

## Methods

### RNA-Seq data

We obtained RNA-Seq data for the ENCODE Tier 1 and Tier 2 cell types mapped against the latest human genome assembly (hg19) using the UCSC track download portal [31, 32]. Only samples from the ENCODE category *long RNA extracts* (>200 bp) were considered, as short RNA extractions aim at identifying small non-coding RNAs while our proviral remnants of interest are considerably longer (mean length of 928 nt). We further restricted the considered tracks to whole cell extracts, as we are interested in the overall analysis of HERV expression in the entire human cells rather than in individual compartments.

We focused on data produced by either the Cold Spring Harbor Laboratories (CSHL) or the California Institute of Technology (Caltech) (Table 1) because these provide the most comprehensive coverage of (mostly) the same cell types, facilitating a direct comparison of the results produced by these two groups. Moreover, due to the fact that Caltech performed both single-end and paired-end sequencing on a subset of cell types, expression analysis results can also be compared between different library preparations. However, the single-end data sets contain fewer reads and we therefore investigated whether or not this lower coverage is still sufficient to detect HERVs, which make up only a small fraction of the cell’s transcriptome.

**Table 1:** ENCODE RNA-Seq data used in this study

Cell type	Tissue	Condition		Number of replicates		
			Tier	CSHL paired-end	Caltech single-end	Caltech paired-end
A549	Epithelium	Cancer	2	2	-	-
B-cells CD20+	Blood	Normal	2	2	-	-
GM12878	Blood	Normal	1	2	2	2
H1-hESC	ESC	Normal	1	2	2	4
HUVEC	Blood vessel	Normal	2	2	2	2
HeLa-S3	Cervix	Cancer	2	2	2	2
HepG2	Liver	Cancer	2	2	2	2
IMR90	Lung	Normal	2	2	-	-
K562	Blood	Cancer	1	2	2	2
MCF-7	Breast	Cancer	2	2	-	3
Monocytes CD14+	Monocytes	Normal	2	2	-	-
SK-N-SH	Brain	Cancer	2	2	-	-

### HERV annotation

We used the currently most comprehensive collection of annotated HERVs, the HERVd database [21, 22]. This database contains 98,008 entries describing 224 different HERV families, from full-length proviral elements to singular Long Terminal Repeats (LTRs). Since the HERVd annotation is based on the hg17 assembly of the human genome we transferred all genomic coordinates to hg19 using the liftOver tool [27].

A number of HERVd entries did not survive the lifting process: 2,342 entries are completely or partially deleted and another 24 entries are split in the latest hg19 assembly. We nevertheless attempted to identify the location of these entries in hg19 by sequence similarity searches using BLAT [33]. Similarity hits were accepted as the origin of a given HERV if the corresponding alignments were gap free, covered the complete query sequence, and had a minimum sequence identity of at least 98 %. In the same fashion we identified additional viral elements in hg19 by using all known HERV sequences as query and accepting new origins when they met the identity cutoff. It should be noted that we only performed similarity searches in regions without existing HERV annotations to avoid duplicated entries. However, while the initial HERVd already contains HERV loci that are overlapping with each other, we decided against filtering these out in order not to lose HERV annotations.

Our initial HERV data set contained 100,495 locations in hg19. HERVd entries located on chromosome Y were excluded from consideration as this chromosome is not covered by all ENCODE datasets used in our study. This filtering step left us with a total of 98,998 annotated HERV loci (Additional file 2) for which we obtained read counts.

### HERV expression in ENCODE RNA-Seq

**Fig. 7:**
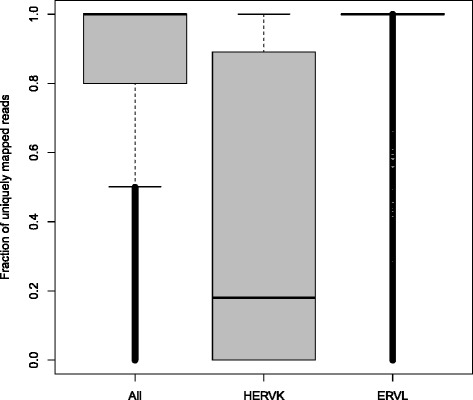
Fraction of uniquely mapped reads. The left box shows the fraction of reads uniquely mapped to HERV loci across all ENCODE samples. The middle box contains the same values for members of the recently integrated HERVK family. The right box shows the family members of ERVL, a very old HERV family

We calculated the read coverage over the HERVd entries for every RNA-Seq experiment using the “featureCount” tool of the subread package [34]. For every annotated viral element it returns the number of reads mapped to it in every analyzed sequencing run. The program was executed with the “primary” option, which forces featureCount to only take primary alignments into account, thus avoiding biased expression values through non-uniquely mapped reads. This is extremely important when working on HERV loci, as the different families, due to their varying integration ages, have unequal degrees of similarities among the family members. Thus, it is easier to assign a read to its exact loci for older and more distinct families than for younger families. An alternative to using the primary alignment would be to only use uniquely mapped reads, but this would bias the expression values towards older HERV families (see Fig. 7).

Furthermore, in the data sets comprised of paired-end reads the entire fragment was counted only once to maintain comparability with the single-end samples. Reads in stranded datasets were counted in a strand-specific manner; these include the single-ended Caltech samples and all CSHL samples.

### Differential expression analysis

The coverage depth of HERV loci between 25 ENCODE samples (Table 1) was compared using the R bioconductor package *DESeq* [35–37] , which is specially designed for differential expression analysis. To achieve a better comparability between samples we normalized their count data by library size and carried out a variance stabilizing transformation based on the inherent biological variability between the replicates of the same condition. We then performed a principal component analysis (PCA) and calculated the Euclidean distances between the transformed expression values to detect overall differences between the samples.

The following analyses were limited to the paired-end RNA-Seq data as PCA revealed extensive differences between single- and paired-end library preparations. Hence to avoid introducing a bias in the differential expression analysis, we excluded single-end data. The read count value of every condition, normalized by the library size, was compared in a pairwise fashion against every other condition, resulting in 171 differential expression analyses and the corresponding fold changes. The *DESeq* implementation of the negative binomial test was than used to find significant differences in the calculated expression values. The initial p-values were adjusted for multiple testing using the Benjamini-Hochberg procedure [38] . In order to identify significantly differentially expressed HERVs we filtered for loci whose absolute logarithmic fold change was at least one and whose adjusted p-Value did not exceed 0.001 (which is equivalent to a false discovery rate of 0.1 %).

For every analyzed cell type, we compiled a list of HERV loci up-regulated in at least one of the pairwise comparisons. By considering the corresponding families of loci, we sought to identify HERVs that are particularly active in certain cell types and under certain conditions.

### Validation on housekeeping genes

In order to ascertain that the differences in HERV expression between different conditions and library preparations reported in this study are not due to computational or experimental biases specific to endogenous viral elements, we repeated our analysis with a set of housekeeping genes. For this purpose we used the list of 3,804 genes compiled by Eisenberg and Levanon [39]. This list was created based on RNA-seq data from 16 different human tissues by first identifying housekeeping exons, i.e. those exons expressed in all tissues, displaying low variance between tissues, and showing no exceptional expression in any single data set. Housekeeping genes were then defined as those genes, for which at least one annotated RefSeq transcript has more than half of its exons classified as housekeeping. When acquiring the annotation file for the housekeeping genes from the UCSC genome browser, only 3,801 entries could be retrieved, as three identifiers [RefSeq: NM_032937, NM_003926, NM_032560] had been removed from the RefSeq database. The assessment of coverage for every housekeeping gene in all our selected ENCODE data sets was carried out exactly as described above for HERVd, including normalization and principal component analysis (The featureCount output for all housekeeping genes can be found in Additional file 3).

## Additional files

Additional file 1:
**Read statistics per sample.** For all analyzed ENCODE samples the number of fragments/reads which are used as input for featureCount are listed as well as the fraction of reads mapping to HERVd annotated loci. (CSV 1 kb) Additional file 2:
**HERVd annotation of HERV loci in hg19.** Tabular file contains columns for unique identifier, chromosome, start and stop position, strand and HERV family, respectively. The remaining columns represent the read counts from featureCount for all of the analyzed ENCODE samples. (CSV 14292 kb) Additional file 3:
**Read counts of housekeeping genes.** Tabular file containing the featureCount counts for all genes classified as housekeeping by Eisenberg and Levanon [39]. (CSV 1000 kb)
